# Simultaneous Carbamazepine and Phosphate Removal from a Moving-Bed Membrane Bioreactor Effluent by the Electrochemical Process: Treatment Optimization by Factorial Design

**DOI:** 10.3390/membranes12121256

**Published:** 2022-12-12

**Authors:** Khanh-Chau Dao, Yung-Pin Tsai, Chih-Chi Yang, Ku-Fan Chen

**Affiliations:** 1 Department of Civil Engineering, National Chi Nan University, Nantou Hsien 54561, Taiwan; 2 Department of Health, Dong Nai Technology University, Bien Hoa 810000, Dong Nai, Vietnam

**Keywords:** advanced oxidation process, electrochemical, factorial design, carbamazepine, moving-bed membrane bioreactor

## Abstract

Pharmaceutical and personal care products are frequently used in various fields and released into water bodies from the outlets of wastewater treatment plants. These products can harm the environment and human health even at low concentrations. Carbamazepine (CBZ), the most persistent pharmaceutical, has frequently been found in surface waters that bypassed the secondary treatments of conventional activated sludge. In addition, the treatment of phosphate in wastewater by the electrochemical process has recently attracted much attention because of its ability to remove, recover, and prevent environmental problems associated with eutrophication. This study proposes using the electrochemical process as an advanced oxidation process to simultaneously treat CBZ and phosphate from the moving-bed membrane bioreactor effluent. The study includes a long-term survey of CBZ treatment efficiency and common parameters of synthetic wastewater in the moving-bed membrane bioreactor system. Afterward, the electrochemical process is applied as an advanced oxidation process for the simultaneous removal of CBZ and phosphate from the moving-bed membrane bioreactor. Under the investigated conditions, CBZ has proven not to be an inhibitor of microbial activity, as evidenced by the high extent of chemical oxygen demand and nutrient removal. Using a factorial design, the electrochemical process using Pt/Ti as anode and cathode under optimal conditions (reaction time—80 min, bias potential—3 V, and electrode distance—1 cm) resulted in as high as 56.94% CBZ and 95.95% phosphate removal, respectively. The results demonstrated the ability to combine an electrochemical and a moving-bed membrane bioreactor process to simultaneously remove CBZ and phosphate in wastewater.

## 1. Introduction

Pharmaceutical and personal care products (PPCPs) are now widely used in various industries and areas of life [1]. The pharmaceuticals used in human medicine are excreted through the urine and feces, where they could be transformed into glucuronides or sulfates, or even remain unchanged [2]. The release of PPCPs from wastewater treatment plants can cause potential harm to the environment and human health, even at low concentrations [3]. Hospital wastewater is considered the largest source of PPCPs emissions. These pollutants are transported into conventional wastewater treatment plants, which lack the necessary mechanisms to remove biorecalcitrant compounds [4,5,6] that can be removed through biological wastewater treatment, between 30 to 60% [7]. Carbamazepine (CBZ), which is one of the most common micropollutants in surface waters [8], is a drug for controlling grand mal and psychomotor epilepsy and is also effective for the treatment of trigeminal neuralgia and bipolar depression [9]. It could be harmful to health due to serious toxic effects on the liver and the hematopoietic system [10].

The activated sludge and membrane separation processes are combined in a membrane bioreactor (MBR). The reactor works the same way as a conventional activated sludge reactor but without secondary clarification requirements [11]. MBR operations provide several advantages, including increased biomass concentrations for efficient chemical oxygen demand (COD), suspended solids, nutrients, and pathogen removal, reduced hydraulic retention time (HRT), and less production of excess sludge [12,13,14]. Previous research has shown that depending on operating conditions and PPCP characteristics, PPCPs removal from MBR processes ranged from none to total elimination [15]. In the early 1990s, the moving bed biofilm reactor (MBBR) was introduced, which uses small plastic carriers in constant motion inside the reactor [16] with the growth of biomass as a biofilm and suspended flocs [17]. The moving bed membrane bioreactor (MBMBR), which combines the MBR and the MBBR, has not only proven effective in eliminating PPCPs [18,19] but also may increase system performance and reduce membrane fouling caused by suspended particles [20]. Besides improving the biomass concentration associated with increased surface area for biofilm formation, carriers in the MBMBR could provide a favorable environment for the activity of both aerobic and anoxic microorganisms [21].

Recently, advanced oxidation processes (AOPs) have been attracting much attention to address persistent pollutants. AOPs generate highly active and reactive hydroxyl radicals (OH*) (E^0^ = 2.8 V/SHE), which can help persistent organic pollutants mineralize faster. As an AOP, the electrochemical process (EC) has been developed [22], which involves the in situ production of OH* radicals through the oxidation and reduction reactions at the anode and cathode by using an external power supply [23]. EC has attracted interest because of its ability to mineralize highly refractory pharmaceuticals [24,25,26]. In general, organic pollutants are oxidized by direct and indirect oxidation. Direct anodic oxidation occurs on the anode at relatively low potentials, before oxygen evolution [27], which involves direct charge transfer reactions between the anode surface and the organic contaminants. In particular, the first step is the anodic discharge of water [28], which produces hydroxyl radicals that are then absorbed on the active sites of the electrode (*M*):

(1) $$H_{2}O\to M\left(OH*\right)+H^{+}+e^{-}$$

In the second step, the absorbed hydroxyl radical oxidizes the pollutant (*R*):

(2) $$M\left(OH*\right)+R\to M+RO+H^{+}+e^{-}$$

where *RO* stands for the oxidized pollutant that hydroxyl radicals can produce continually.

Mixed metal oxide (MMO) electrodes, known commercially as Dimensionally Stable Anodes (DSA), are made of corrosion-resistant basic materials like tantalum or titanium coated with a layer of metal oxides such as RuO_2_, IrO_2_, or Pt. They have “active” behavior and low overpotential for oxygen evolution, exhibiting refractory organic compounds’ partial and selective oxidation. In contrast, others (e.g., SnO_2_, PbO_2_) with high oxygen evolution have “non-active” behavior and exhibit a higher efficiency in oxidizing organic compounds in wastewater. MMO electrodes have mainly been used to treat pollutants in the presence of chloride due to their low capacity to oxidize and mineralize organics [27,29].

Indirect oxidation is achieved by in situ electro-generation of highly oxidant species (Cl*, Cl_2_, HClO, and ClO^−^) in a bulk solution such as active chlorine when the chloride is electrolyzed [30,31] under the following chemical reactions:

(3) $$2Cl^{-}-2e\to Cl_{2}$$

(4) $$Cl_{2}+H_{2}O\to HOCl+Cl^{-}+H^{+}$$

(5) $$\begin{array}{l} HOCl\to OCl^{-}+H^{+} \end{array}$$

Moreover, the EC has also proved an effective method for phosphate removal by water electrolysis close to the cathode, producing a local high pH when the oxidation of the water generates H^+^, neutralizing the OH^−^ produced on the cathode [32] under the following reactions:

(6) $$\mathrm{Cathode}:\;\begin{array}{l} 4H_{2}O+4e^{-}\to 4OH^{-}+2H_{2}↑ \end{array}$$

(7) $$\begin{array}{l} 5Ca^{2+}+3HPO_{4}{}^{2-}+4OH^{-}\to Ca_{5}{\left(PO_{4}\right)}_{3}OH↓+3H_{2}O \end{array}$$

(8) $$\mathrm{Anode}:\;2H_{2}O\to 4H^{+}+O_{2}↑+4e^{-}$$

As a result, the cathode surface is the only place where calcium phosphate precipitates. Because of the high local pH, calcium phosphate precipitation could proceed effectively in alkaline, neutral, and acidic solutions even when buffers were present [33,34]. The precipitation of phosphate, in this case, involves one or more calcium phosphate salts, including CaHPO_4_ 2H_2_O, CaHPO_4_, Ca_4_H(PO_4_)_3_.3H_2_O, Ca_3_(PO_4_)_2_, Ca_5_(PO_4_)_3_OH, and amorphous calcium phosphate. Removing the phosphate from the wastewater by the EC could provide three benefits: phosphorus recovery, prevention of environmental problems associated with eutrophication, and wastewater reuse [35].

The main aim of the present study was to evaluate the efficiency of the combination of MBMBR and EC processes in removing persistent CBZ drug and nutrients. The study included a long-term survey of CBZ treatment efficiency and common parameters of synthetic hospital wastewater in the MBMBR system. After that, the optimization process by factorial design (FD) using Pt/Ti electrodes was applied to find the optimum operating conditions in the EC process to enhance CBZ and phosphate removal simultaneously.

## 2. Materials and Methods

### 2.1. Experimental Set-Up

#### 2.1.1. Moving Bed Membrane Bioreactor System

**Figure 1:**
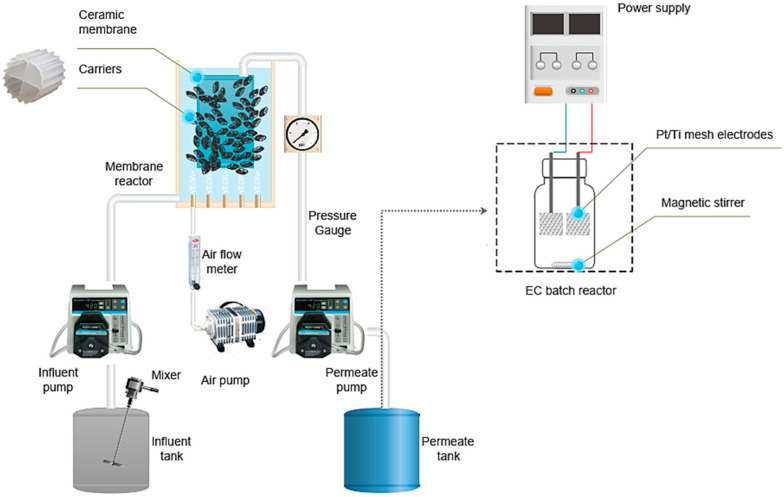
Schematic diagram of the MBMBR–EC experimental set-up.

A Moving Bed Membrane Bioreactor (MBMBR) system (25 L volume) with a flat-sheet membrane module (GVE Environmental Co., Ltd., Taoyuan, Taiwan), a pore size of 0.1 µm, and an effective area of 0.25 m^2^ was vertically fixed in the reactor, Figure 1. To relax the membrane module, the membrane module was operated for 8 min on and 2 min off. To keep the water level constant, the influent flow rate was changed to match the effluent flow rate. Timers automatically controlled the systems. Air diffusers were placed at the bottom of the reactor for aeration and to support carriers moving inside the reactor and controlled by an airflow meter. Twenty percent of the volume of the carriers (GVE Environmental Co., Ltd., Taoyuan, Taiwan) was added to the reactor. The dimensions of the carriers were 10 × 10 mm, the density was >0.96 g/cm^3^, and the effective specific surface area was >850 m^2^/m^3^.

The MBMBR was fed with synthetic wastewater simulating hospital wastewater to provide a source of carbon, nutrients, and trace metal ions for supporting biomass growth. Two peristaltic pumps controlled the system (Masterflex L/S, Vernon Hills, IL 60061, USA) for influent and permeate. The oxygen concentration in the reactor was controlled online (DC-5110 DO Transmitter, Suntex, New Taipei City, Taiwan). After 20–30 days of operation, the membrane module was removed, washed with tap water, and immersed in sodium hypochlorite solution (2000 mg/L) for 2 h. Before being reinstalled in the system, the membrane was rewashed with water.

The experimental plan had two different phases. In phase I, the system was operated for 28 days for the start-up process. The activated sludge seed was collected from a conventional wastewater plant at National Chi Nan University, Taiwan, and added to the reactor to reach a 5000 mgTSS/L concentration with an MLSS/MLVSS ratio (mixed liquor volatile suspended solids to mixed liquor suspended solids) of 0.78. The oxygen concentration (DO) was controlled to maintain the concentration in the range of 1.5–2.0 mg/L; HRT and solids retention time (SRT) were maintained at 1 day and 25 days, respectively. In phase II, long-term investigation, to achieve nutrient removal, DO was set up in the range of 2.5 to 3.0 mg/L, and the maximal lactate steady state concentration (MLSS) was controlled in the range of 3000–4000 mg/L. Experiments were carried out at ambient temperature.

#### 2.1.2. Electrochemical System

The EC reactor was undivided, with a capacity of 100 mL. A set of two rectangular 1mm thick 1 µm platinised titanium meshes, with total dimensions of 6.25 cm^2^, connected in parallel with an alternate electrode acting as the anode and the other as the cathode, were immersed into the wastewater in the reactor. The inter-electrode gap was 1 cm. The electrodes were purchased from William Gregor Ltd. (London, United Kingdom). The reactor was placed on a magnetic stirrer with a magnetic bead at the bottom to ensure continuous mixing of the solution. The EC process was started by switching on the DC power supply. Before conducting each EC experiment, the electrodes were thoroughly washed using distilled water.

### 2.2. Chemicals and Synthetic Wastewater

Oasis HLB 3 cc Vac cartridges were purchased from Waters, Milford, MA, USA. HPLC-grade chemicals, including acetonitrile, acetone, ethyl acetate, and methanol, were supplied by Sigma-Aldrich. Before using them in the HPLC, all the solvents were filtered through 0.45 μm membrane filter paper (Millipore, Merck, Darmstadt, Germany) and degassed ultrasonically for 30 min.

CBZ was obtained from Sigma-Aldrich (purity ≥ 99.0%). A Milli-Q water purification system (resistivity of 18.2 MΩ cm at 25 °C) produced ultra-pure water. Stock solutions containing 1 g/L of CBZ were prepared in the Milli-Q water from powdered substances and kept in the dark at 4 °C to prevent degradation.

Analytical-grade chemicals were used to prepare synthetic wastewater, Table 1. This resulted in concentrations of chemical oxygen demand (COD) 272.26 ± 45.75 mg/L, NH_4_^+^-N 51.82 ± 10.44 mg/L, PO_4_^3−^-P of 15.61 ± 0.6 mg/L. The wastewater was prepared twice a week and stored in a 100 L storage tank under mixing conditions. In the storage tank, due to the slight sedimentation and degradation of the organic materials, there were fluctuations in chemical oxygen demand (COD) and in the NH_4_^+^-N and PO_4_^3−^-P concentrations in the MBMBR influent. The CBZ was added into the synthetic wastewater at the range of 100 μg/L.

### 2.3. Analytical Methods

Inlet and outlet samples were collected three times a week and stored frozen until the analysis. COD, MLSS, PO_4_^3−^-P, total solids (TS), volatile solids (VS), MLSS, and MLVSS were determined according to the standard methods for the examination of water and wastewater [36]. The colorimetric method in the presence of potassium dichromate with an absorbance of 600 nm using a UV-Vis spectrometer (DR 5000, Hach, CO, USA) was applied to determine the COD when the indophenol method was used to measure NH_4_^+^-N [37]. To measure the TS, 100 mL of the sample was collected from sludge liquor and dried at 105 °C for 24 h when VS was measured at 550 °C for 30 min.

The amount of biomass fixed in the carriers was measured by collecting 14 carriers from the reactor and putting them in a beaker with 200 mL of deionized water. After being sonically treated for 20 min to wash out the biomass fixed in the carriers, the suspension was dried and weighed [38].

Solid-phase extraction (SPE) was developed to detect the CBZ in the wastewater. The inlet samples were filtered using a 0.45 μm glass-fiber filter (Millipore, Merck, Darmstadt, Germany) when the permeate samples were not further filtered. Next, the CBZ was extracted from the 50 mL water samples using Oasis HLB 3 cc Vac cartridges. For SPE procedures, the solid-phase adsorbent was preconditioned prior to loading the samples with 5 mL of methanol and then 5 mL of Milli-Q water. The samples were then passed through the cartridge at a rate of 5 mL/min. In the following step, five 1 mL aliquots of ethyl acetate-acetone (50:50, *v*/*v*) were used to elute the cartridge at a flow rate of 1 mL/min. The combined aliquots were then gently evaporated with high-purity nitrogen flow before being redissolved in 1 mL of methanol. The Agilent 1200 HPLC used for the CBZ detection was equipped with a G1315D diode array detector and a G1316A column oven (Agilent Technologies Co., Ltd., Santa Clara, CA, USA). The column temperature was set to 30 °C, and the wavelength for detection was 210 nm. For separation, an Eclipse XDB-C18 column (4.6 × 150 mm, 5 μm particle size, Agilent) was used. For the mobile phase, acetonitrile/water (31:69, *v*/*v*) was used at a flow rate of 1 mL/min. The volume of injection was 20 μL.

The removal efficiency of the CBZ, COD, ammonia, and phosphate was calculated:

(9) $$C\%=\frac{C_{in}-C_{out}}{C_{in}}\times 100$$

where *C_in_* and *C_out_* represent the inlet and outlet concentrations, respectively.

### 2.4. Design of Experiments and Optimization

To examine the EC process, several factors, such as reaction time, bias potential, and electrode distance, are to be studied. However, it takes a lot of time and effort to study every single factor. To overcome these difficulties, factorial design (FD) can reduce the number of experiments and obtain process optimization [39,40]. Moreover, this determines the effect of each factor on response and the interaction effects of the factors [40,41]. In 2-level FD, the factors are set in two levels and called “high” and “low” or “+1” and “−1,” respectively.

This study’s optimization goals include finding process parameters (reaction time, bias potential, and electrode distance) to maximize the CBZ and phosphate removal rate using Design Expert 11.0.0 (trial) to predict the outputs. Table 2 presents the factor coding and the variable ranges.

## 3. Results and Discussion

### 3.1. Carbamazepine Removal in MBMBR

**Figure 2:**
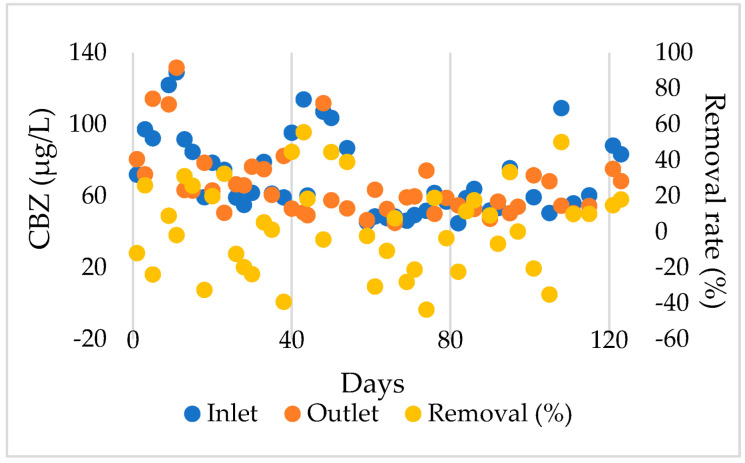
CBZ evolution in MBMBR system.

The high recalcitrance of CBZ was proved by poor CBZ removal effectiveness in the MBMBR with a maximum efficiency of ∼50%, frequently lower than 20% (Figure 2). In addition, the experiment results showed that the concentrations of CBZ inlet (54.88 to 129 µg/L) and outlet (ranges from 49.11 to 131.52 µg/L) fluctuated to a great degree. As a result, the removal efficiency also fluctuated accordingly. On some experimental days, the CBZ concentration in the outlet was higher than in the inlet. This phenomenon can be explained as the release of accumulated CBZ in sludge [42,43]. Toward the end of the operation, the inlet and outlet CBZ concentration values tended to become more stable, reflecting the stable operation of the MBMBR.

**Figure 3:**
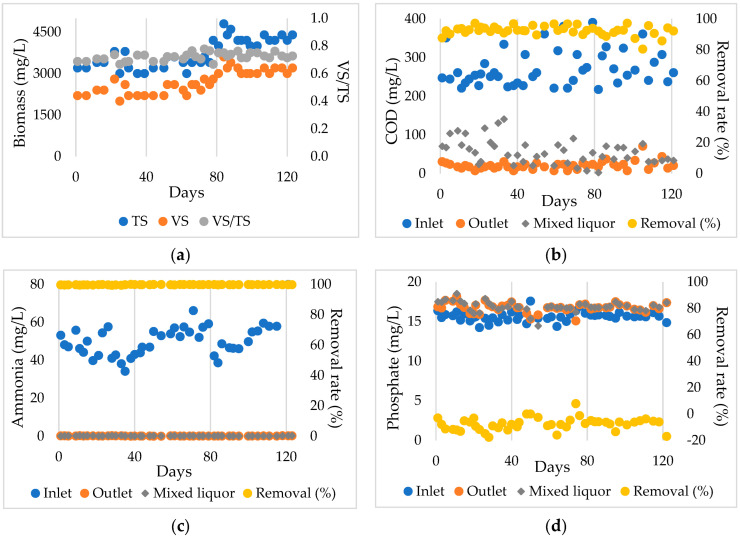
The evolution of biomass (**a**), COD (**b**), ammonia (**c**), and phosphate concentration (**d**) in MBMBR.

This study clearly shows that CBZ is ineffectively removed by the MBMBR. The previous study showed that CBZ removal efficiency tends to be better in anoxic than in aerobic conditions [44,45]. Furthermore, the removal of micropollutants by MBR-based systems, in general, is limited because of the large pore size of the membrane module and the low MLSS concentration [46,47,48]. In addition, the properties and ratio of carriers used in the reactor also play an important role in enhancing CBZ removal in MBMBR systems [49]. In this work, the amount of MLSS maintained inside the reactor and biomass attached to the carriers was not high, Figure 3. Therefore, this could partly be attributed to the low removal of CBZ. The previous study showed that the hydrophobicity of CBZ, which partly favors its sorption, and its resistance to biodegradation could be responsible for its poor removal efficiency [50]. However, it could adsorb to the sludge, extending its retention time in the reactor for subsequent biodegradation [51].

### 3.2. Biological Removal Efficiencies in MBMBR

After 28 days of acclimation, the MBMBR was operated for 120 days continuously for long-term investigation. The system shows stable operation through stability in TS, vs concentration, and VS:TS ratio, which remained stable between 0.67 and 0.73 throughout the experiment (Figure 3a). Furthermore, regardless of the change in MLSS concentration, the system showed high efficiency in removing TS, as demonstrated by the non-detection in the outlet.

After the acclimation period, the attached biofilm on the carriers was about 0.2 g TS/L. Then the attached biofilm densities tended to increase gradually during operation. At the end of the operation cycle, the biofilm attached rose slightly to about 0.55 g TS/L. A lower concentration of attached biofilm densities was shown compared to the previous study [52]. This could be explained by the aeration process and low organic loading rate in the reactor [52]. Additionally, the organic matter in the inlet, which remained relatively steady throughout the study, had no direct relation to the biofilm.

The inlet COD concentration was 272.26 ± 45.75 mg COD/L, while the COD concentration in the outlet and the mixed liquor maintained 21.17 ± 10.97 mg/L and 56.43 ± 31.73 mg/L, respectively, Figure 3b. The high removal efficiency (92.37 ± 3.16%) and the insignificant difference between COD concentrations in the output and mixed liquor showed that the CBZ had not inhibited microbial activity as sulfonamide under the same concentration range investigated [19].

The inlet and outlet of ammonia concentration (NH_4_^+^-N) were 51.82 ± 10.44 mg/L and 0.14 ± 0.06 mg/L, respectively, representing 99.72 ± 0.13% of removal efficiency (Figure 3c). The removal of ammonia from the system could be explained by the oxidation of the ammonia to nitrate by nitrifying bacteria and its conversion to biomass inside the reactor. In addition, the long SRT could be responsible for developing long-generation-time microorganisms such as autotrophic nitrifying bacteria [53].

The inlet and mixed liquor of phosphate concentration (PO_4_^3−^-P) was 15.61 ± 0.6 mg/L and 16.88 ± 0.65 mg/L, respectively (Figure 3d), and after the treatment, the outlet concentration increased to 16.75 ± 0.58 mg/L, which shows the ineffectiveness in removing phosphate. This could be under aerobic conditions, with phosphate-accumulating organisms (PAOs) not being able to grow well, leading to poor phosphate removal [54,55].

### 3.3. Optimization of the EC Process

#### 3.3.1. Preliminary Investigations

**Figure 4:**
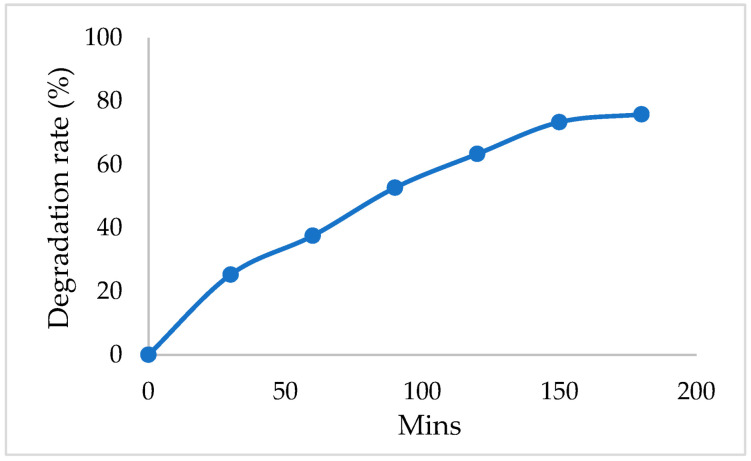
Preliminary test for CBZ degradation in the EC process with Pt/Ti electrodes (electrode distance: 1cm, potential: 3V).

To simultaneously enhance the removal of CBZ and phosphate, MBMBR effluent was proposed to combine with EC. Figure 4 shows the preliminary result of the CBZ degradation in the EC process with Pt/Ti electrodes. The removal efficiencies of the CBZ reached about 80% within 150 min of the EC process from the MRMBR effluent.

#### 3.3.2. Design of Experiments by Factorial Design (FD)

A sum of 24 experimental runs, including 3 replicates of each, was conducted for optimization based on 2^k^ FD. Table 3 presents the actual and predicted percentages of CBZ and phosphate removal. The experiments were conducted randomly to determine the influence of factors on the responses.

#### 3.3.3. Analysis of Variance (ANOVA)

The main effects, the interacting factors affecting CBZ and phosphate removal, were measured by ANOVA. The results of the ANOVA for the two responses are listed in Table 4. Each factor’s importance is quantified by the sum of squares (SS), and as the SS value rises, so does the importance of that factor in the process. *p* values < 0.05 are used to identify the potential significance for each main and interaction effect [56].

#### 3.3.4. Main and Interaction Effects

F-value and *p*-value were used to calculate the significance of each coefficient when the coefficient of determination (R^2^) was used to measure the proportion of total variability explained by the model and should be at least 0.8 [57]. From Table 4, the value of R^2^ was reasonably close to 1 (0.9936), implying that the model explained around 99.36% of the variability in the new data, while the model’s F-value and *p*-value were 356.63 and < 0.0001, respectively. This confirmed that the estimated model adequately described the experimental data. It is clearly shown that, among factors, A (reaction time), B (bias potential), C (electrode distance), and the interactions between AB and BC were the most influential factors on CBZ removal when AC and ABC were less important. The previous study also indicated that reaction time and current density were more important than other factors in CBZ removal in the EC process [30].

**Figure 5:**
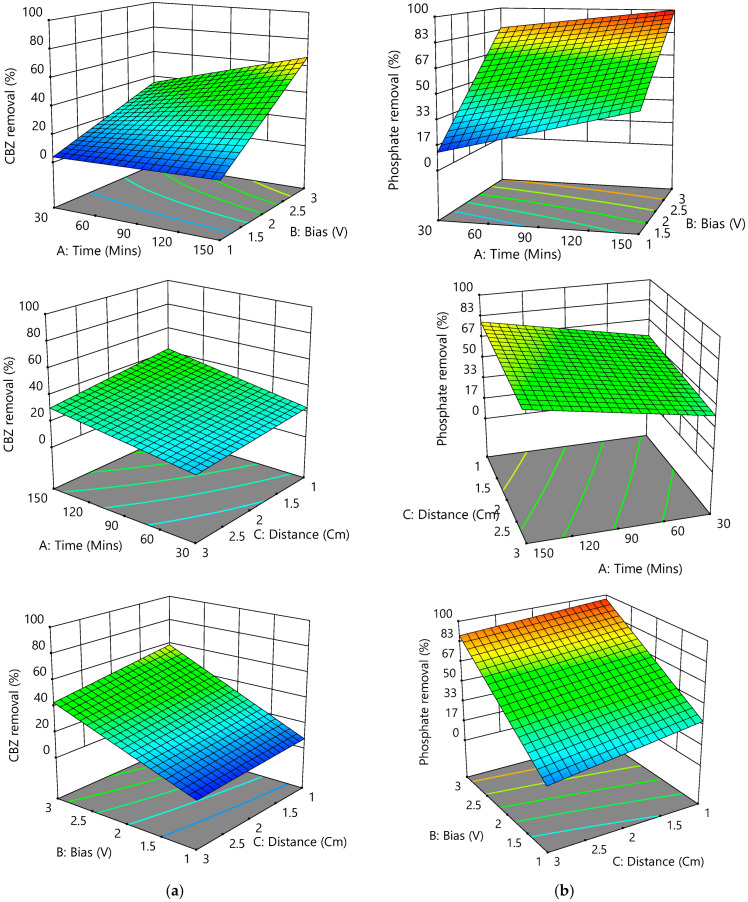
Response surface plots for (**a**) CBZ and (**b**) phosphate removal efficiency.

In the case of phosphate removal, A, B, C, AB, BC, and ABC were the most influential factors when AC was insignificant. In addition, the phosphate removal response had a greater than 80% of confidence level (*p* < 0.05), while the F-value and *p*-value were 850.16 and <0.0001, respectively. The model’s coefficient of determination R^2^ was 0.9973, implying that the estimated model described around 99.73% of the variability in the new data. Removing the phosphate at low current density could be due to the precipitation of calcium phosphate, not adsorption, while the co-precipitation of CaCO_3_ and Mg(OH)_2_ with calcium phosphate was reduced [58]. Figure 5a,b shows a positive effect of A and B on both CBZ and phosphate removal in the EC system, while C shows a slight negative effect. After discarding insignificant terms, the coded equation could be given as:

CBZ removal (%) = 28.26 + 8.61×A + 29.91 × B − 5.04 × C + 7.18 × A × B − 1.33 × A × C − 2.84 × B × C − 1.43 × A × B × C(10)

Phosphate removal (%) = 60.41 + 11.79 × A + 32.11 × B − 6.25 × C − 4.31 × A × B + 2.24 × B × C + 4.7 × A × B × C(11)

#### 3.3.5. Normal Probability Plot of Residuals

**Figure 6:**
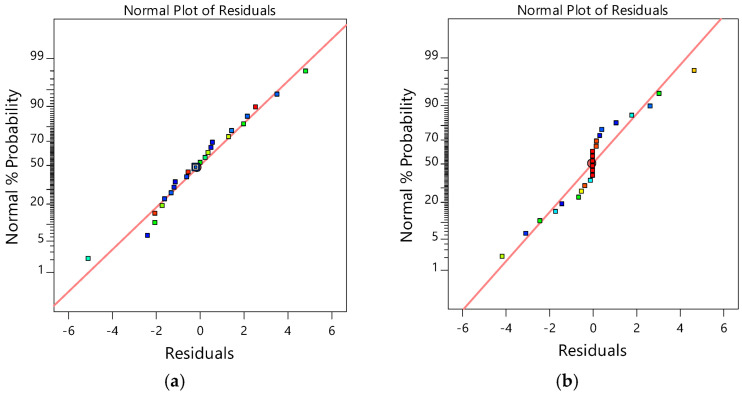
Normal probability plots of residuals for (**a**) CBZ removal and (**b**) phosphate removal.

It is assumed that the observations are normally distributed in the analysis of a two-level FD. Plotting a normal probability plot of residuals is used as the best method to check such normality assumption. The data are normally distributed if the data points on the plot lie reasonably close to the straight line [59,60]. Figure 6 reveals that the experiments come from a normally distributed population because the data points of all the responses were reasonably close to the straight line.

#### 3.3.6. Optimization

**Figure 7:**
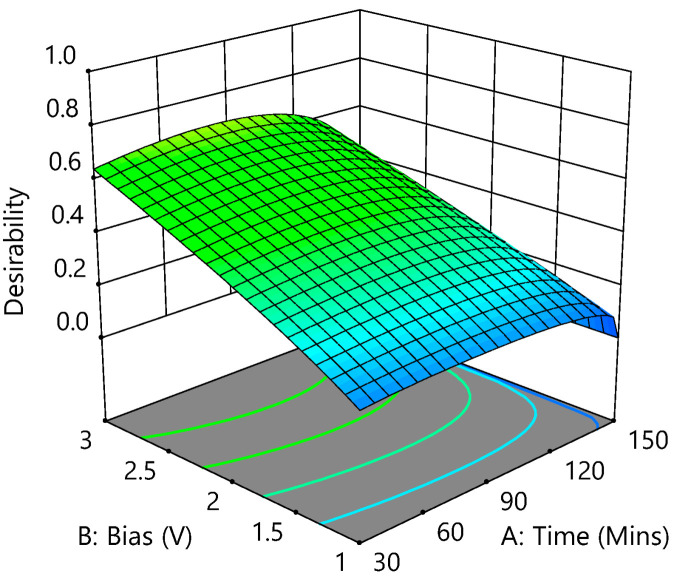
Desirability-fitted 3D surface at an electrode distance of 1 cm.

The CBZ and phosphate removal were optimized by a multiple response method called the desirability (D) function to optimize process parameters such as reaction time, bias potential, and electrode distance. Optimization aimed to find the highest level of CBZ and phosphate removal. To achieve maximum desirability, bias potential and electrode distance were set to within the range. In contrast, reaction time was set to minimum (importance level 2/5); CBZ removal (importance level 5/5) and phosphate removal (importance level 1/5, because of the high removal rate of phosphate) were set to maximum, respectively. Figure 7 shows the numerical optimization’s graphical desirability generated from 58 optimum points.

Among 58 starting points, the best CBZ and phosphate removal rate (56.94% and 95.95%, respectively) was found at reaction time—80 min, bias potential—3 V, and electrode distance—1 cm with 0.693 of desirability.

## 4. Conclusions

The present study investigated the effectiveness of a moving bed membrane bioreactor (MBMBR) with a common carrier for carbamazepine (CBZ) and nutrient removal. Afterward, the factorial design (FD) methodology determined the significant parameters, investigated their interactions, and optimized the electrochemical process (EC) conditions concerning CBZ and phosphate removal.

The MBMBR effectively removed COD and ammonia in this study but was ineffective in CBZ and phosphate removal. The membrane pore size, properties, and ratio of carriers used inside the bioreactor could improve CBZ removal in the MBMBR. Thus, altering other carriers, along with a thicker attached-growth biofilm, could be promising. The results also showed that EC as an AOP could be combined effectively with an MBMBR to treat CBZ and phosphate in wastewater simultaneously.

The most significant factors under investigating conditions could be identified using FD. The EC process effectively improved the simultaneous removal of CBZ and phosphate in the MBMBR effluent, even when the CBZ concentration was as low as ug/L. The bias potential and the reaction time play a more important role than the distance between the two electrodes. Maximum removal of CBZ and phosphate reached 56.94% and 95.95%, respectively, under desired conditions (reaction time—80 min, bias potential—3 V, and electrode distance—1 cm).

## Figures and Tables

**Table 1:** Components of synthetic wastewater used in the study (mg/L).

Component	Concentration
Meat extract	82.5
Peptone	120
NH_4_Cl	143.2
NaCl	5.25
CaCl_2_·2H_2_O	3
MgSO_4_·7H_2_O	1.5
CuCl_2_·2H_2_O	0.03
K_2_HPO_4_·3H_2_O	84
C_6_H_12_O_6_	187.5
NaHCO_3_	825

**Table 2:** Experimental ranges and levels of the factors used in the factorial design.

Factor	Name	Units	Range and Level	
			−1	+1
A	Reaction time	Mins	30	150
B	Bias potential	V	1	3
C	Electrode distance	cm	1	3

**Table 3:** Factorial design matrix of three variables along with experimental and predicted responses for CBZ and phosphate removal.

	Factor	Factor 2	Factor 3	CBZ Removal			Phosphate Removal		
Run	A	B	C	Actual	Predicted	Residual	Actual	Predicted	Residual
1	30	1	1	5.62	6.20	−0.58	15.71	15.29	0.42
2	150	1	1	7.59	8.88	−1.29	55.88	58.30	−2.42
3	150	3	1	75.57	77.60	−2.03	100	100	0
4	30	3	3	25.18	30.26	−5.08	72.86	77.01	−4.15
5	30	3	3	30.51	30.26	0.25	81.68	77.01	4.67
6	150	1	3	2.32	4.69	−2.37	32.31	30.51	1.80
7	30	1	3	2.22	1.63	0.59	10.17	9.09	1.08
8	30	1	1	4.61	6.20	−1.59	17.93	15.29	2.64
9	150	3	1	77.08	77.60	−0.52	100	100	0
10	150	1	1	10.35	8.88	1.47	57.66	58.30	−0.64
11	150	3	3	56.71	56.33	0.38	100	100	0
12	150	3	3	57.65	56.33	1.32	100	100	0
13	30	3	1	40.54	40.51	0.03	92.71	93.06	−0.35
14	150	3	3	54.62	56.33	−1.71	100	100	0
15	30	1	3	2.15	1.63	0.52	9.42	9.09	0.33
16	30	1	1	8.38	6.20	2.18	12.22	15.29	−3.07
17	150	1	3	8.22	4.69	3.53	28.81	30.51	−1.70
18	150	3	1	80.15	77.60	2.55	100	100	0
19	150	1	3	3.53	4.69	−1.16	30.41	30.51	−0.10
20	30	3	3	35.09	30.26	4.83	76.50	77.01	−0.51
21	150	1	1	8.71	8.88	−0.17	61.35	58.30	3.05
22	30	3	1	38.48	40.51	−2.03	93.23	93.06	0.17
23	30	3	1	42.52	40.51	2.01	93.24	93.06	0.18
24	30	1	3	0.52	1.63	−1.11	7.68	9.09	−1.41

**Table 4:** ANOVA results for CBZ and phosphate removal response.

	Source	Sum of Squares	df	Mean Square	F-Value	*p*-Value	
	Model	16,508.91	7	2358.42	356.63	<0.0001	significant
	A-Reaction time	1779.86	1	1779.86	269.14	<0.0001	
	B-Bias	12,598.67	1	12598.67	1905.12	<0.0001	
CBZ removal	C-Electrode distance	608.83	1	608.83	92.07	<0.0001	
	AB	1236.11	1	1236.11	186.92	<0.0001	
	AC	42.45	1	42.45	6.42	0.0221	
	BC	194.26	1	194.26	29.37	<0.0001	
	ABC	48.73	1	48.73	7.37	0.0153	
	Pure Error	105.81	16	6.61			
	Cor Total	16,614.72	23				
	Std. dev.	2.57		R^2^		0.9936	
	Mean	28.26		Adjusted R^2^		0.9908	
	Model	30,134.29	7	4304.90	850.16	<0.0001	significant
	A-Reaction time	3338.69	1	3338.69	659.35	<0.0001	
Phosphate removal	B-Bias	24,747.18	1	24,747.18	4887.25	<0.0001	
	C-Electrode distance	938.63	1	938.63	185.37	<0.0001	
	AB	446.43	1	446.43	88.16	<0.0001	
	AC	11.52	1	11.52	2.28	0.1509	
	BC	120.65	1	120.65	23.83	0.0002	
	ABC	531.19	1	531.19	104.90	<0.0001	
	Pure Error	81.02	16	5.06			
	Cor Total	30,215.31	23				
	Std. dev.	2.25		R^2^		0.9973	
	Mean	60.41		Adjusted R^2^		0.9961	

## Data Availability

The data presented in this study are available on request from the corresponding author.
